# Nanoparticle induced fusion of lipid membranes†

**DOI:** 10.1039/d4nr00591k

**Published:** 2024-04-29

**Authors:** Sofía Blasco, Lukáš Sukeník, Robert Vácha

**Affiliations:** a CEITEC – Central European Institute of Technology Kamenice 5 625 00 Brno Czech Republic robert.vacha@muni.cz; b National Centre for Biomolecular Research, Faculty of Science, Masaryk University Kamenice 5 625 00 Brno Czech Republic; c Department of Condensed Matter Physics, Faculty of Science, Masaryk University Kotlářská 2 611 37 Brno Czech Republic

## Abstract

Membrane fusion is crucial for infection of enveloped viruses, cellular transport, and drug delivery *via* liposomes. Nanoparticles can serve as fusogenic agents facilitating such membrane fusion for direct transmembrane transport. However, the underlying mechanisms of nanoparticle-induced fusion and the ideal properties of such nanoparticles remain largely unknown. Here, we used molecular dynamics simulations to investigate the efficacy of spheroidal nanoparticles with different size, prolateness, and ligand interaction strengths to enhance fusion between vesicles. By systematically varying nanoparticle properties, we identified how each parameter affects the fusion process and determined the optimal parameter range that promotes fusion. These findings provide valuable insights for the design and optimization of fusogenic nanoparticles with potential biotechnological and biomedical applications.

## Abstract 2

We found that the optimal fusogenic nanoparticle is able to form a stalk but does not overstabilize it, facilitating the opening of a fusion pore.

## Graphical abstract

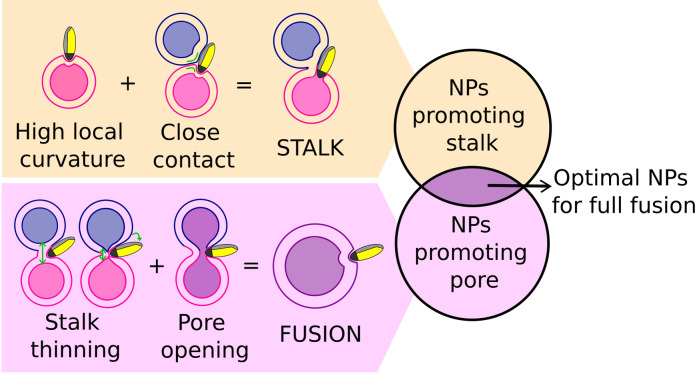

## Introduction

Membrane fusion is a process in which two distinct membranes merge into a single bilayer. Fusion is involved in a number of cellular processes mainly related to the transport of substances between organelles or between separate cells. These substances are usually transported in vesicles that fuse with other membranes to deliver their contents.^1,2^ Membrane fusion is also involved in the infection of membrane-enveloped viruses, which use specific proteins to facilitate the fusion with the host membrane.^3^ Membrane fusion is also a critical step in drug delivery *via* artificial vesicles called liposomes. Fusion of the liposome with the target membrane is necessary for direct delivery of cargo to the cytoplasm. Without fusion, liposomes are internalized *via* endocytosis, which very often leads to their degradation.^4^

Fusion of two membranes is not a spontaneous process, therefore several energy barriers need to be surpassed. Already prior the initiation of the fusion, the hydration repulsion needs to be overcame to allow close contact between the membranes.^5^ Once the membranes are in close contact, the membrane fusion consists of two energy barriers.^6^ The first barrier is related to the formation of a stalk, where lipids from the outer leaflet of the apposed membranes mix, while the inner leaflets remain separate. The second barrier is related to the opening of a fusion pore which involves mixing of inner and outer leaflets and results in a connection between the volumes previously separated by each bilayer. After that, the pore is expanded resulting in complete fusion.^7^

Membrane fusion is carried out by specific proteins that facilitate the process in cells.^1,8^ The role of these proteins can be artificially mimicked by nanoparticles.^9–12^ The design of synthetic nanoparticles that can induce membrane fusion and mimic the effect of fusion peptides could enable targeted and efficient drug delivery to specific cells or organelles. However, the properties that facilitate or induce fusion by such proteins and nanoparticles remain elusive. At the same time, systematic manipulation of nanoparticle properties could elucidate the basic principles of membrane fusion.

In this work, we systematically modified spheroidal nanoparticles and quantified their ability to induce fusion between two vesicles. We focused on the effect of nanoparticle prolateness, size and ligand–receptor binding strength in each step of the fusion process. Using molecular dynamics simulations, we determined the relationship between nanoparticle properties and fusion efficacy elucidating the basic principles of membrane fusion.

## Results

**Fig. 1:**
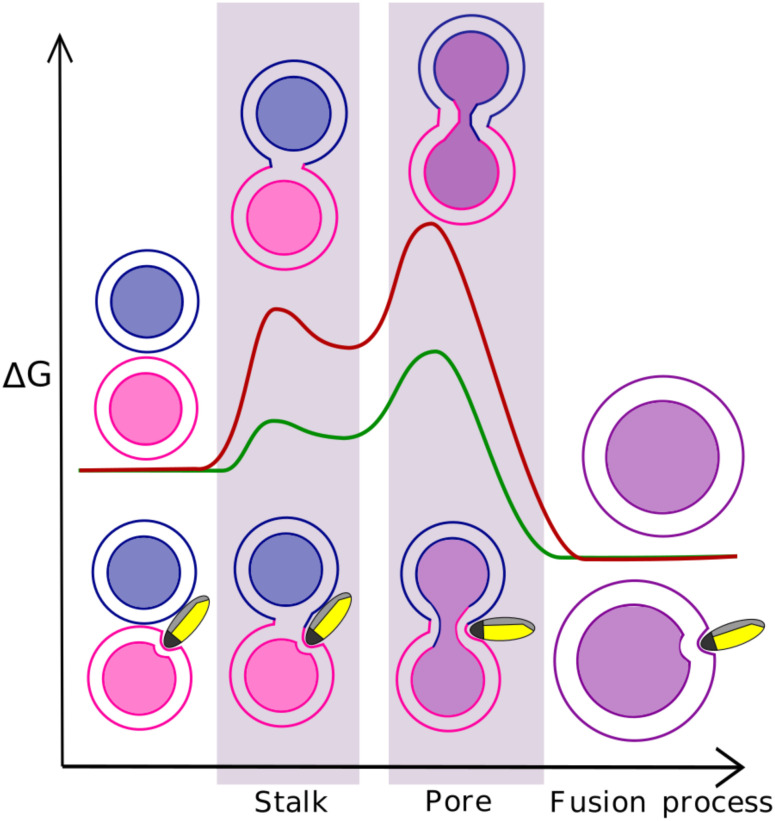
Illustration of the free energy profiles of the fusion process with and without nanoparticle. There are two main energy barriers associated with the stalk formation and fusion pore opening. The nanoparticle properties can effectively lower these energy barriers and facilitate the fusion process.

**Fig. 2:**
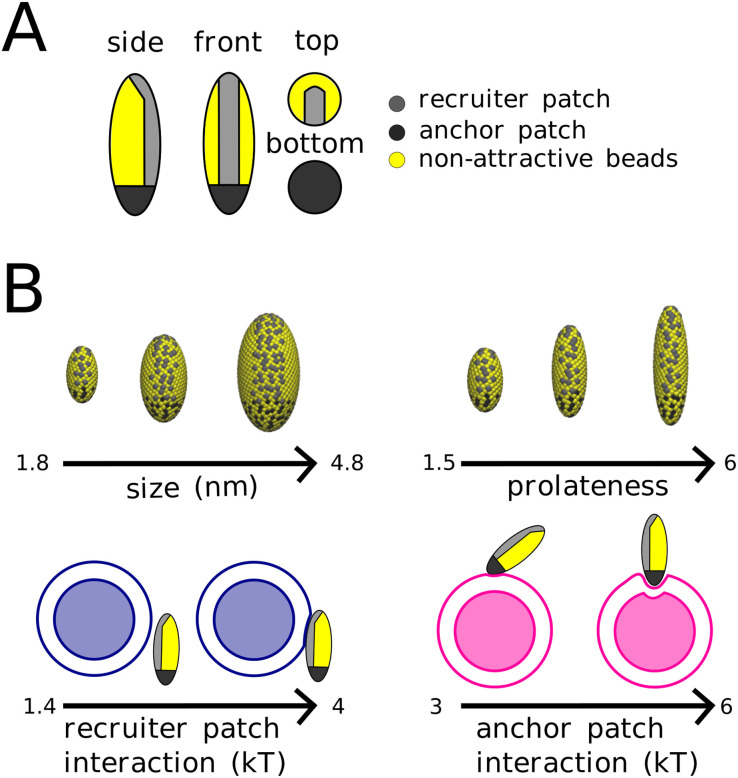
Illustration of the employed model of (A) nanoparticle with ligand covered patches that adhere to lipid receptors and (B) the range of explored parameters – nanoparticle size and prolateness and ligand–receptor interaction strength.

We performed molecular dynamics simulations to design a nanoparticle which lowers the energy barriers associated with the fusion process, as depicted in Fig. 1. We investigated spheroidal nanoparticles, characterized by their equatorial and polar radii, and their aspect ratio 
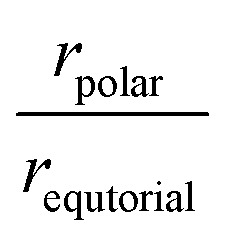
 known as prolateness, denoting any nanoparticle as [equatorial diameter (nm), polar length (nm)]. The nanoparticle had two ligand patches: an anchor patch and a recruiter patch, each consisting of different ligands, as illustrated in Fig. 2. To elucidate the relationship between nanoparticle properties and fusion efficacy, we systematically varied the strength of receptor–ligand interactions for both patches, nanoparticle prolateness, and nanoparticle size. Equatorial radius varied from 1.8 nm to 4.8 nm and prolateness from 1.5 to 6, which results in nanoparticle dimensions ranging from [3.6 nm, 5.4 nm] to [9.6 nm, 28.8 nm]. For recruiter patch, the interaction strength of ligands varied from 1.4 to 4 kT, while for anchor patch it varied from 3 to 6 kT. The combination of these parameters yielded 3200 nanoparticle variants, each simulated in 10 independent replicas. We used Cooke–Deserno lipid model, in which lipids are represented by three beads.^13^ This highly coarse-grained model allowed us to simulate many nanoparticle variants with reasonable computational cost. In summary, we evaluated the fusion efficacy of various nanoparticles by performing over 32 000 molecular dynamics simulations of membrane fusion between two vesicles induced by the nanoparticle.

**Fig. 3:**
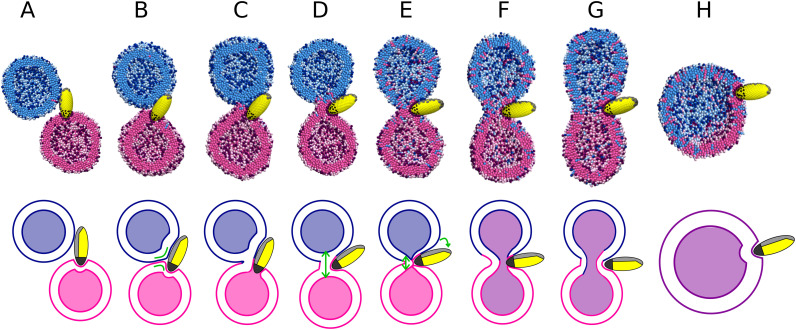
Simulation snapshots of the fusion process induced by nanoparticle with schematic representation below. (A) Binding of the two vesicles to a nanoparticle, one vesicle per ligand patch. (B) Close contact between the vesicles with a high local curvature. Green lines in schematic highlight the high curvature of the vesicles at the contact point before stalk formation. (C) First hydrophobic contact between the vesicles. (D) Vesicles with a formed stalk. Contents of vesicles are still separated. (E) Recruiter patch unbinding and nanoparticle rotation at the stalk. The green arrows show nanoparticle rotation and point to the thinning of the stalk. (F) Anchor patch unbind from one side of the stalk and fusion pore opens. Vesicle contents are connected. (G) Pore expansion. (H) Fully fused vesicle.

We designed our nanoparticles to lower the energy barriers associated with the fusion process. The first barrier consists of hydration force,^5^ and low accessibility of lipid tails, whose splay initiate the stalk formation.^14,15^ The hydration force can be overcome by forcing close contact between the membranes, while for increasing the accessibility of lipid tails, a highly curved membrane is essential.^14,16^ Although the lipid splay observed in more detailed simulations cannot be represented with the employed 3-bead lipid model, the increased accessibility of lipid tails resulting from a high curvature still initiates the stalk formation. In this case, a part of lipid tail is exposed from membrane core enabling its interaction with the opposing membrane and thus bridging the hydrophilic gap between the membranes. Therefore, the anchor patch was placed at the tip of the nanoparticle, which induces high local membrane curvature in the bound vesicle, see Fig. 3B. The recruiter patch was designed to recruit the second vesicle from solution and move it towards the anchor patch, as shown in Fig. 3A. This patch thus covers one side of the nanoparticle from one tip of the nanoparticle to the beginning of the anchor patch (see Fig. 2). With this design of nanoparticle patches, both vesicles are brought together at a point where their membranes are highly curved, and thus promote the formation of stalk (see Fig. 3C and D).

After stalk formation, we observed two necessary steps for the nanoparticle to open a fusion pore. The first step consists on the unbinding of the recruiter patch. This step causes the nanoparticle to rotate and reorient it at the stalk, leading to thinner stalk, see Fig. 3E. This thinning is similar to what happens in SNARE-mediated fusion.^17^ Once the stalk is thin enough, the second step to open the fusion pore is observed around the anchor patch. In the stalk the anchor patch penetrates through the membrane in the middle of the stalk where it interacts with the inner leaflet of the opposite membrane. After the recruiting patch has unbound, the anchor patch interaction needs to be released for the pore to open, see Fig. 3F. Once there is fusion pore, its expansion into a fully fused vesicle is almost instantaneous.

### Stalk formation

**Fig. 4:**
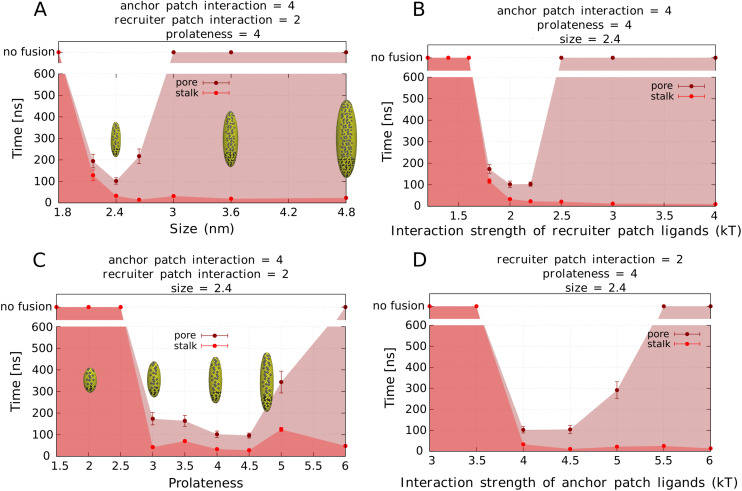
Half-lifes of time needed for stalk formation and times needed for vesicle fusion as a function of nanoparticle parameters. For all parameters there is a threshold above which stalk forms, and an optimal region for which stalk and fusion pore (and therefore fusion) occurs. Optimum parameters are: equatorial radius (size) of 2.4 nm, interaction of recruiter patch of 2 kT, prolateness of 4 and interaction of anchor patch of 4 kT. The time scales were estimated by comparing the diffusion coefficient of a simulated vesicle with its calculated value, and we obtained the equivalence of 1 ns per 30 000 timesteps.

To promote stalk formation, the nanoparticle needs to generate sufficient local curvature. The local curvature is promoted by the anchor patch, with the highest curvature achieved when the anchor patch is fully wrapped by the vesicle. Stronger membrane binding induces a higher curvature, therefore the nanoparticle requires a strongly interacting anchor patch of at least 4 kT per ligand. However, this effect plateaus once the anchor patch is fully wrapped (*i.e.*, for interactions ≥4 kT), as shown in Fig. 4D. Lower interaction values resulted in the nanoparticles lying flat on the membrane, due to incomplete patch wrapping, see Fig. S3.† The nanoparticle size and prolateness also alter its ability to induce curvature in the bound membrane. Nanoparticles with too low prolateness (≤2) and small size (equatorial *r* ≤ 2.4 nm) have an anchor patch which is not big enough to induce sufficient membrane curvature, see Fig. S1.† The recruiter patch interaction is more relevant for the binding of second vesicle to the nanoparticle. The minimum interaction strength of recruiter patch required to bind a vesicle varied between 1.6 and 2 kT, depending on the nanoparticle dimensions (see Fig. S1† for dimension-specific interaction values). Considering that the recruiting patch size is proportional to the nanoparticle size, larger nanoparticles have more ligands in their patches, therefore they require lower interaction strengths for vesicle binding. In summary, nanoparticle promoting stalk formation should have a strongly interacting anchor patch (above 4 kT per ligand), a recruiter patch interaction strength at least around 2 kT, and it needs to be sufficiently big and prolate: nanoparticles bigger than [4.8 nm, 14.4 nm].

### Pore formation

Pore formation and consequent complete fusion was only achieved by a small fraction (about 1/5) of the nanoparticles within the simulated timescales. As mentioned above, the unbinding of the recruiter and anchor patches determines whether the vesicles remain in the stalk intermediate or progress to pore opening and fusion. The recruiter patch is able to unbind for interaction strengths of the recruiting patch below 2.5 kT and nanoparticle sizes under 3 nm, see Fig. 4A and B. Overall stronger interaction of nanoparticles (stronger ligand–receptor interaction and/or nanoparticle with more ligands) prevents the unbinding and thus stabilizes the stalk intermediate (step C and D of Fig. 3). Variation of the prolateness (within the studied range) does not affect this fusion step, because longer nanoparticles are still able to unbind their recruiting patch and reorient in the stalk. The anchor patch is able to unbind from second membrane inside the stalk for interaction strengths below 5.5 kT and prolateness below 6, see Fig. 4C and D. Stronger interacting anchor patches thus remain in the stalk and reduce pore opening. Nanoparticles with increasing prolateness have a longer anchor patch in our model, leading to the deeper nanoparticle penetration and stronger interaction in the stalk. Therefore, more ligands are able to interact with the opposite membrane on the stalk, preventing anchor patch unbinding. Failing to unbind the anchor patch from the stalk results in a stable stalk intermediate, preventing pore opening and completing the fusion, see Fig. 3. Smaller, less prolate, and weaker interacting nanoparticles thus are able to fuse once there is a stalk.

In general, for each parameter there is an optimal range in which both stalk formation and pore opening are facilitated.

### Consecutive fusion

We additionally investigated whether the nanoparticles were able to fuse vesicles consecutively. Indeed, we found that after fusion of two vesicles, nanoparticles could induce fusion with a new vesicle, see Fig. S5.† The mechanism/pathway of fusion remained the same and the final state of the nanoparticle on the fused vesicle again remained as before fusion, which suggest that even an additional vesicle could fuse. However, note that fusion was observed only in 4 out of 10 simulation replicas and the time needed to fuse was about twice the time needed for the first vesicle. In the cases where fusion did not happen, stalk formation occurred but it disappeared after some time. After dissolution of the stalk, the nanoparticle anchor patch was not completely wrapped anymore, preventing any new stalk formation (Fig. S5†).

### Fusion efficacy

**Fig. 5:**
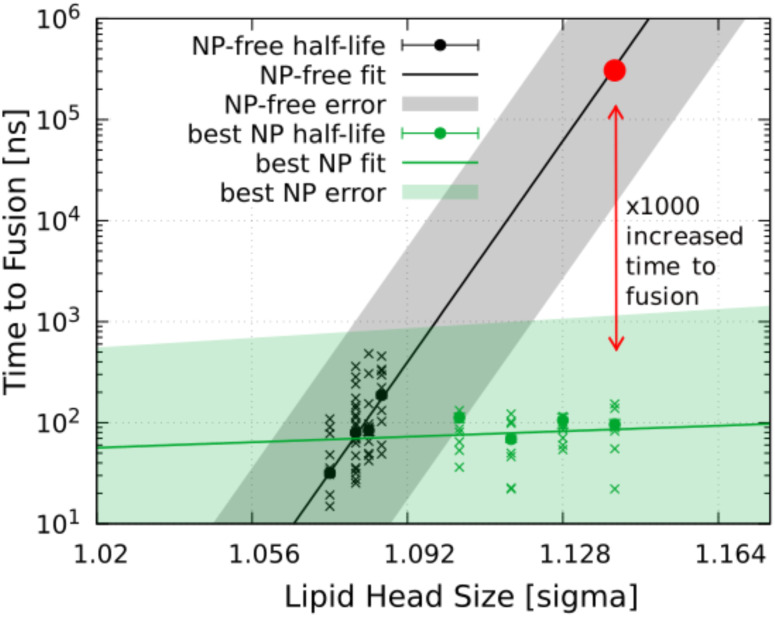
The time to fusion between two vesicles with lipid heads of varying size. The crosses represent the time to fusion from the individual simulation replicas, and the full circles the time to fusion half-life. We compare systems without and with the fusogenic nanoparticles. The time to fusion is shown in logarithmic scale. The times were fitted (shown by the continuous line) to approximate the effect of the nanoparticle, which enhanced the fusion about 1000 times for standard head size of 1.14 nm.

To determine the efficacy of our fusogenic nanoparticles, we compared its effect (the time to fusion) to systems without the nanoparticle, a nanoparticle-free system. In nanoparticle-free systems, we decreased the size of the lipid heads, which was shown to enable stalk formation due to increased availability of lipid tails.^18^ In Fig. 5, we compare the time to fusion in systems with and without nanoparticle. The figure shows that the time to fusion increases rapidly with increasing lipid head size in the nanoparticle-free system, while it increases less (is more independent) when the nanoparticle is present. We used this system to extrapolate the time to fusion in vesicles with standard lipid head-group size of 1.14 nm (0.95*σ*, as described in Deserno–Cooke model^19^), which suggests that the nanoparticle produces fusion about 1000 times faster than nanoparticle-free fusion.

### Alternative pathways

Note that in few cases, we observed fusion following a different path, in which the nanoparticle is internalized by the vesicles *via* membrane pore. For this path the optimal parameters are different. Nanoparticles of equatorial radius larger than 3.6 nm induced fusion *via* this path as depicted in Fig. S4.†

### Four bead model

The robustness of our findings was demonstrated using a different lipid model. We performed simulations using four-bead lipid model,^20^ which has higher membrane thickness, slower lipid flip-flop rate and reduced propensity for pore formation compared to three-bead model. All of those properties are expected to make membrane fusion more difficult.^21,22^ Therefore, we reduced lipid head size to 1.08 nm to facilitate the fusion to timescales accessible within our simulations.

The nanoparticle parameters that promote fusion in this model differ slightly from those observed using the 3-bead model. The interaction strength of both anchor and recruiter patch need to be higher to produce the same membrane perturbations as in the 3-bead model. The anchor patch strength is increased to 5 kT to adopt the upright position and be completely wrapped, while the recruiting patch requires at least 2.5 kT to be able to bind the second vesicle. Nanoparticles with prolateness higher than 2 are not inducing fusion with the 4-bead model because the vesicles were not able to completely wrap the anchor patch and thus did not create the necessary curvature to form the stalk. Interestingly, the fusion path with this model differs in the lack of the penetration of the nanoparticle tip in the stalk, see Fig. S6.† Therefore, the interaction of the anchor patch is not responsible in this case for the opening of the fusion pore. Also, this indicates that the nanoparticle penetration is not necessary for fusion. These findings confirm that fusion with our design of nanoparticle is possible in a membrane with suppressed lipid flip-flop, demonstrating robustness of our findings with the 3-bead model (with high flip-flop rate).

For completeness, we also tested the 4-bead model without the fix that suppresses flip-flop. We were able to see fusion also with this model. As in the 4-bead model with suppressed flip-flop, the interaction strength needed in both patches was 5 kT (anchor patch) and 2.5 kT (recruiter patch). In contrast, we observed fusion with standard head group size of 1.14 nm and with the same size and prolateness parameters as in the 3-bead model (size 2.4 nm, prolateness 4).

## Discussion

Molecular dynamics simulations have been readily used to study the mechanisms of membrane fusion both in the presence of proteins or peptides^15,22–24^ or in protein-free systems.^6,9,26–28^ Several previous studies have shown that it is possible to use nanoparticles to promote membrane fusion.^9–12^ Most of these studies use functionalized gold nanoparticles. Though these nanoparticles can induce stalk formation, they are not capable to promote pore opening by themselves, and they use external factors such as addition of calcium^9,10^ or heating of the nanoparticle^12^ to continue the fusion process. Silica nanoparticles have also been used for membrane fusion, and while they can facilitate stalk and hemifusion diaphragm formation, they do not catalyze the formation of a fusion pore.^11^ In contrast, we study spheroidal nanoparticles because of their higher curvature inducing capability, and we rely solely on the nanoparticle properties to facilitate fusion. It was unclear how different nanoparticle properties affect the fusion including different steps of the fusion process. The work presented here fills these gaps by evaluating the fusion induced by spheroidal nanoparticles of various properties.

For two vesicles to fuse, they initially need to overcome the repulsion caused by hydration layer on top of each membrane^5^ and their lipid tails need to be accessible to other vesicles.^25^ Positive curvature of membrane or negative intrinsic curvature of lipids facilitate the tail accessibility and thus promote the initial step of fusion, leading to the formation of the fusion stalk.^7,16,18^ After stalk formation, the next step is the formation of a fusion pore between vesicles. The pore is initiated by the stalk thinning.^23^ In biological systems with cells and viruses, membrane fusion is facilitated by fusion proteins which are able to perform the necessary steps from initiating vesicle contact to full fusion.^3,29^

In our system with nanoparticle-induced vesicle fusion, the fusion process is facilitated by the nanoparticle properties. The prolate shape and location of the ligand patches ensure the close contact between the vesicles, and produce the membrane curvature needed for the lipid tails to be accessible. Once the stalk is formed, the nanoparticle rotation and reorientation perpendicular to the stalk causes the stalk to become thinner. Finally, nanoparticle detachment from the opposite leaflet of the stalk (caused by penetration of the nanoparticle tip through the membrane), results in the opening of the fusion pore. We found the fusion stalk when nanoparticles were above a certain size, prolateness and ligand–receptor interaction strength. Subsequently, opposite nanoparticle properties were required for stalk thinning and formation of fusion pore. The overlap in the parameters that promoted stalk formation and pore opening resulted in the optimal parameters to induce complete vesicle fusion.

We additionally demonstrated the robustness of our findings by also observing nanoparticle-induced fusion with the 4-bead lipid model, which adds a fourth bead to each lipid and penalizes lipid flip-flop. The 3-bead model has a low energy barrier of flip-flop which may underestimate the energy barrier of pore formation. Therefore, using a model with suppressed flip-flop should model pore formation correctly. We successfully observed fusion with the 4-bead model, demonstrating that our results with the three-bead model are not due to the higher flip-flop rate of the 3-bead model.

Nanoparticle-induced fusion can be useful for applications in the field of drug delivery. Liposome drug delivery is applied in fields like cancer therapy^30,31^ or vaccine delivery.^32^ Extracellular vesicles can also be used in similar way.^33^ However, there are some limitations of liposome delivery such as lack of specific targeting or inefficiency in content delivery to the cytoplasm. The inefficiency is caused by endosomal degradation because the liposomes are usually internalized *via* endocytosis.^4^ The ideal delivery of liposome-based carriers would be to target the desired cell and fuse with its cytoplasm membrane delivering its contents directly to the cytoplasm. Combining liposomes with fusogenic nanoparticles could be a way of achieving this. Nanoparticles could be also used for the modification of extracellular vesicles, because after fusion both the content of the vesicle and its lipid composition could be changed.^34^ In fusion with biological membranes, the surface of the membrane is populated by a variety of molecules such as carbohydrates or proteins. Therefore, the distance necessary to overcome to start the fusion process will be higher than for artificial membranes like liposomes. It is estimated that the distance at which fusion processes start in biological membranes is about 10–20 nm.^35^ Therefore, fusion proteins or fusogenic nanoparticles need to be large enough to be able to interact with both membranes and start the fusion process.

Finally, we briefly discuss the possibility of experimentally developing such a nanoparticle. Currently, there exist many methods^36–38^ for synthesis of nanoparticles with various sizes and shapes, including spheroidal nanoparticles.^39^ Gold nanoparticles are commonly studied due to their non toxicity and stability, which makes them attractive candidates for use in biomedical applications. The sizes of such nanoparticles can be as small as 1 nm and can be functionalized in a number of ways. Conjugation with PEG, amino acids, RNA or folate are some examples of common functionalization.^40^ It is also possible to add the functional groups in patches, as shown in ref. 41, or to have nanoparticles composed of several metals, whose organization can be ordered or random.^42^ Therefore, it seems that the technology necessary to develop fusogenic nanoparticles similar to the investigated ones could be available.

## Conclusions

We have demonstrated that spheroidal nanoparticles covered with ligands can induce spontaneous fusion of vesicles. The efficacy of induced fusion depends on the nanoparticle properties and we showed the optimal parameters – window of nanoparticle, size, prolatness, and interaction strength. For our nanoparticle design, we found the optimal parameters to be: an equatorial radius (size) of 2.4 nm, interaction of recruiter patch of 2 kT, prolateness of 4 and interaction of anchor patch of 4 kT, which results in a nanoparticle of dimensions [4.8 nm, 19.2 nm]. These findings provide valuable insights not only for the design and optimisation of liposome-based drug delivery systems but also for *in vitro* applications of membrane fusion such as modification or loading of extracellular vesicles/liposomes.

## Methods

All simulations were performed using LAMMPS,^43^ with the use of Langevin thermostat.^44^ Temperature was set to 1 kT and timestep to 0.01*τ*. Center of mass motion of the entire system was eliminated using the option “zero yes”. Additionally, “gjf yes” option was turned on applying the Gronbech–Jensen/Farago time-discretization^45^ of the Langevin model to enable longer timesteps, while still producing the correct Boltzmann distribution of atom positions. The viscous damping term was set to 100*τ*.

We employed Deserno lipid model, an implicit-solvent coarse-grained model, in which the phospholipid molecules are represented by three-bead chains.^13^ The first bead represents the hydrophilic headgroup, and is purely repulsive, while the other two beads represent hydrophobic tails and attract each other. Half of the lipids had a modified headgroup bead, with an additional attractive interaction towards nanoparticle ligands, to act as simplified model receptors. The excess of receptors was chosen to eliminate the effects of receptor diffusion. We simulated vesicles of 10 nm radius.

The spheroidal nanoparticles were composed of three types of beads, with a radius of 1.2 nm, the same as phospholipid tail beads. The body of the nanoparticle is composed of purely repulsive, *i.e.* hydrophilic, beads. The other two bead types represent nanoparticle ligands with an additional attractive interaction with the membrane receptors. Each nanoparticle had two patches of ligands, each binding to receptors belonging to different vesicles. One patch was covering the tip (anchor patch), and the other patch was along one side of the longitudinal surface (recruiting patch). The ligand patches location is depicted in Fig. 2. All beads interacted *via* a Weeks–Chandler–Anderson repulsive potential.^46^ Tail–tail and ligand–receptor interactions additionally interacted *via* a cosine squared attractive potential.^13^

For each nanoparticle, we varied size (corresponding to the equatorial radius), prolatenes (corresponding to the aspect ratio) and ligand–receptor binding strength. The size was varied between 1.8 and 4.8 nm, the prolatenes was varied between 1.5 and 6, the ligand strength of the recruiting patch was varied between 1.4 and 4 kT and the ligand strength of the anchor patch was varied between 3 and 6 kT. An overview of the nanoparticles and their parameter variation is shown in Fig. 2.

The simulation protocol consisted of three steps. In the first step, we equilibrated a single vesicle for 100 000 timesteps. In the second step, we placed the equilibrated vesicle and the nanoparticle together so that the anchor patch was close to the vesicle surface. We equilibrated this system for 1 000 000 timesteps, which was enough for the anchor patch to be completely enveloped by the vesicle. Finally, in the last step, we added the second vesicle near the recruiting patch and simulated the system for up to 20 000 000 timesteps, see Fig. S2.†

To assess the fusogenic ability of the nanoparticles, we evaluated the fusion between the two same vesicles without a nanoparticle. In this case, the vesicles were kept close to each other to allow their interaction *via* an harmonic potential around them. However, vesicles with head size 1.14 nm which we used in the simulations with the nanoparticle, did not fuse within the simulated time, so we reduced the size of the head group between 1.068 and 1.092 nm to enhance fusion. The fusion half-life depends exponentially on the size of the headgroup. Therefore, by determining the fusion half-life for 1.068 and 1.092 nm, we can estimate the fusion half-life at 1.14 nm, see Fig. 5. This allowed us to determine the effectiveness of our nanoparticles in promoting fusion compared to nanoparticle-free fusion of the same vesicles.

The pore formation barrier of fusion may be underestimated by the Deserno model due to its low barrier for lipid flip-flop^20,47,48^ For this reason, we validated our results using a four bead lipid model,^20^ which adds a hydrophobic bead to lipid tail and penalizes lipid flip-flop between leaflets. This model represents a thicker membrane with a higher barrier of lipid flip-flop in comparison with the three-bead model. Therefore, the 4-bead model should model this aspect of fusion more correctly.

### Analysis

We performed 10 independent simulations of each nanoparticle–vesicles system. We had around 3200 systems, which makes the total number of around 32 000 simulations. From each simulation, we calculated the time to stalk formation and the time to fusion. The time to stalk formation and the time to fusion have exponential distribution, so we evaluated them *via* their half-life, which we calculated from the cumulative distribution function.

To analyse when a stalk is formed, we analyzed the point when lipid tails of both vesicles become in hydrophobic contact. Two tails were considered to be in contact if their centers of mass were within 3.3 nm of each other (*σ* times tail-cutoff defined by Deserno model).

We consider two vesicles to be fused from the moment their contents connect *via* a fusion pore, *i.e.* the moment when a pore is detected, provided it is maintained for the rest of the simulation.

To approximate the timesteps of the simulations, we calculated the diffusion coefficient of a single vesicle, and compared it to its expected diffusion coefficient. This yielded about 1 ns per 30 000 timesteps.

## Author contributions

S.B. carried out the molecular dynamics simulations and analyzed the data with help of L.S. R.V. and L.S. designed the research. S.B., L.S., and R.V. wrote the article.

## Conflicts of interest

The authors declare no competing interests.

## Supplementary Material

NR-016-D4NR00591K-s001
