# Supplementary material for: Nanoparticle induced fusion of lipid membranes
Source: Nanoscale. 2024 Apr 29;16(21):10221–9. doi: 10.1039/d4nr00591k (PMC11138393; doi:10.1039/d4nr00591k)
Supplement: NR-016-D4NR00591K-s001 [file NR-016-D4NR00591K-s001.pdf]

Electronic Supplementary Material (ESI) for Nanoscale.
This journal is © The Royal Society of Chemistry 2024

Supporting Information

Nanoparticle induced fusion of lipid membranes

Sofia Blasco,[†,‡] Lukáš Sukeník,[†,¶] and Robert Vácha[*,†,‡,¶]

[†] CEITEC – Central European Institute of Technology, Masaryk University, Kamenice 753/5, 625 00 Brno, Czech Republic

[‡] National Centre for Biomolecular Research, Faculty of Science, Masaryk University, Kamenice 753/5, 625 00 Brno, Czech Republic

[¶] Department of Condensed Matter Physics, Faculty of Science, Masaryk University, Kotlářská 267/2, 611 37 Brno, Czech Republic

[*] E-mail: robert.vacha@muni.cz

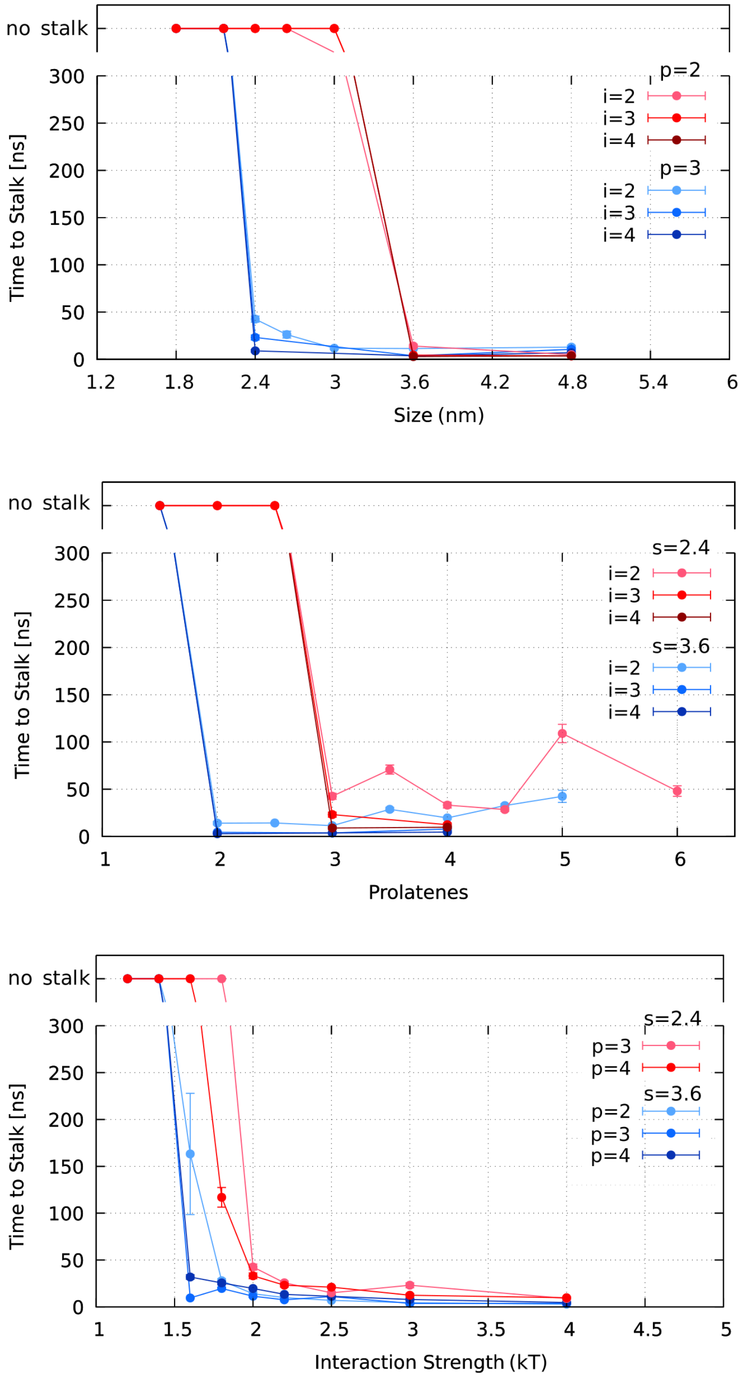

Figure S1: Times to stalk formation of different prolate nanoparticles. The figure shows the time to stalk depending on the variation of each of the nanoparticle parameters: size, prolateness and interaction strength. The three parameters follow the same trend, with differences depending on the other two parameters.

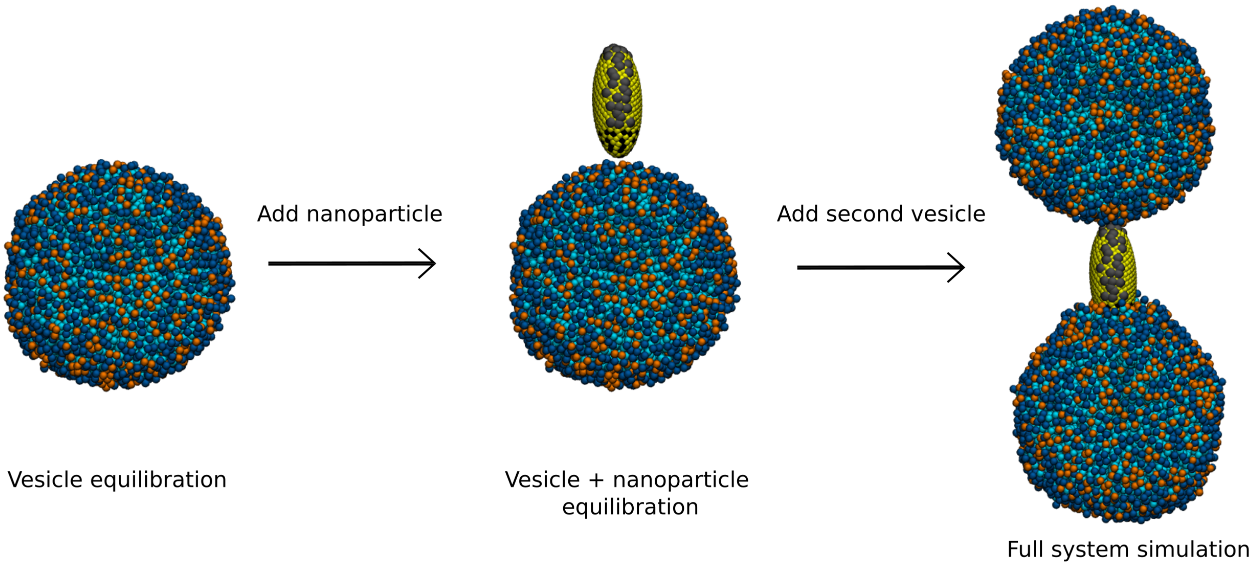

Figure S2: The simulation protocol was divided in three steps. First, vesicle only equilibration (100 000 timesteps), followed by vesicle and nanoparticle equilibration (1 000 000 timesteps), and finally the production run with both vesicles and the nanoparticle (20 000 000 timesteps). In each step, we added the vesicles near the corresponding ligand patch to enable their binding.

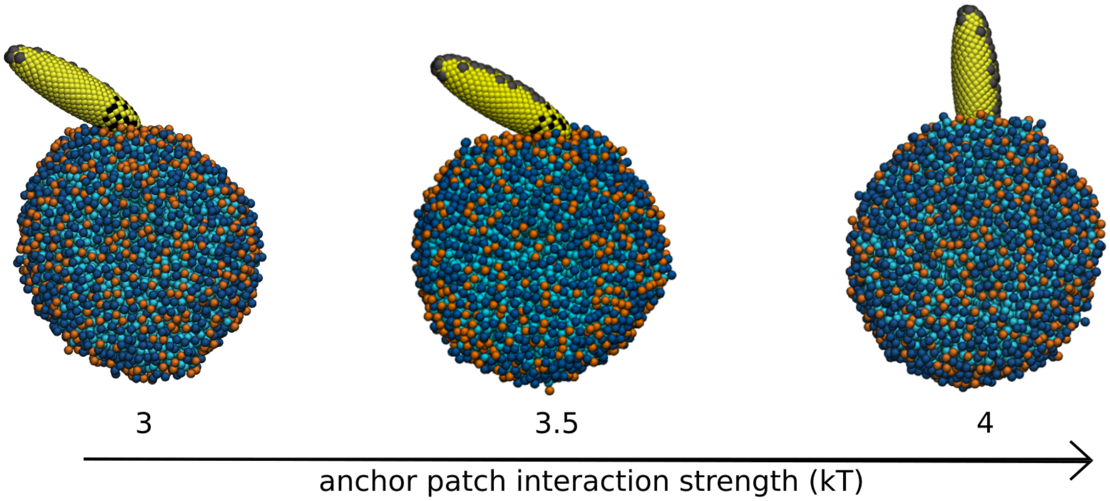

Figure S3: Nanoparticle anchor patch wrapping depending on the interaction strength of the ligands. The figure shows a nanoparticle of size: 2.4 nm and prolateness: 4. Only when the interaction strength is up to 4 kT the anchor patch is completely wrapped by the vesicle, otherwise it lay flat on top of the membrane.

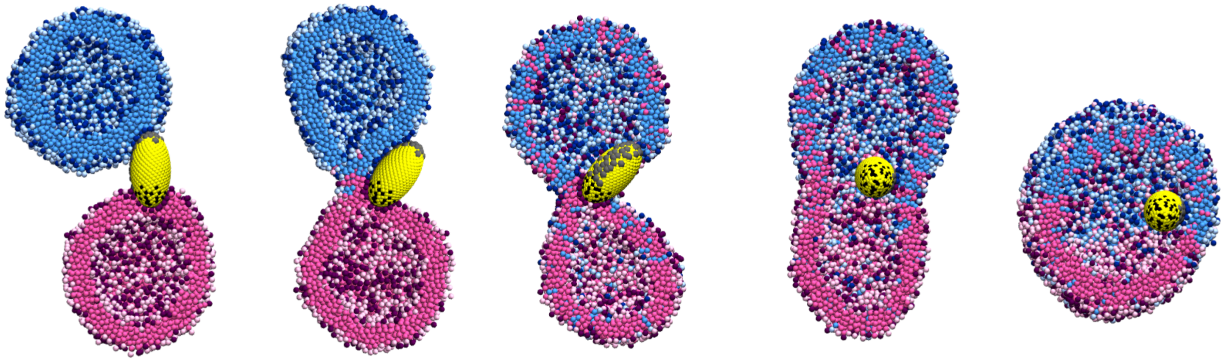

Figure S4: Alternative fusion pathway followed by some nanoparticles of size 3.6 nm in which the nanoparticle is internalized instead of remaining outside of the vesicles.

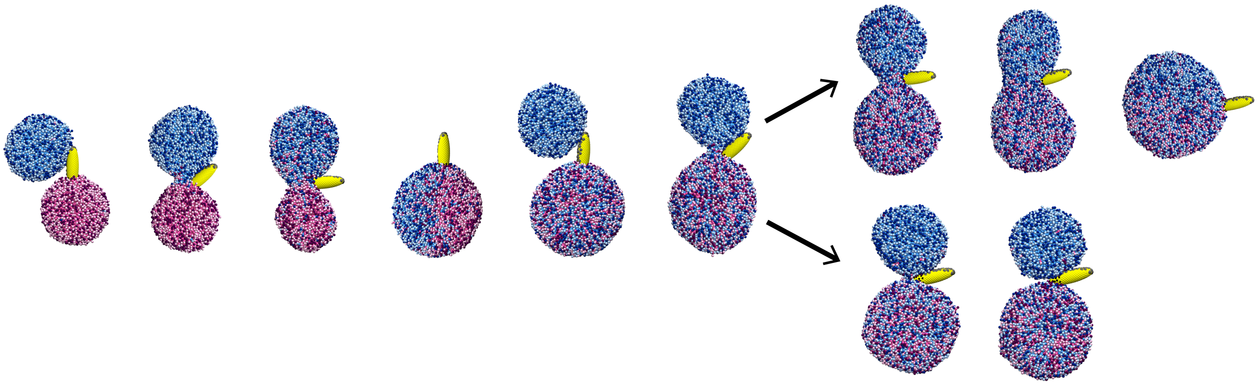

Figure S5: Consecutive fusion of two vesicles with another vesicle. After stalk formation, there is two possible outcomes. Either the vesicles fuse as they do with the first vesicle, or the stalk becomes unstable.

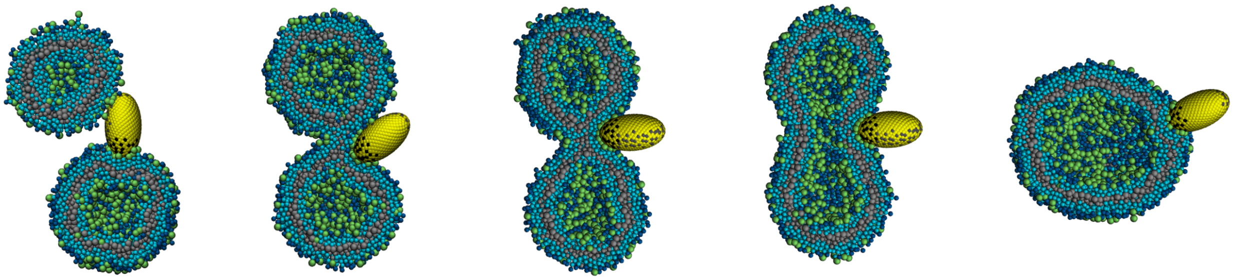

Figure S6: Nanoparticle induced fusion between vesicles simulated with the 4-bead lipid model. This lipid model prevents the penetration of the nanoparticle tip through the membrane (as it occurred when using the 3-bead model), but still the fusion pore is able to open between the vesicles.
